# Performance of the Mayo Risk Score in Predicting Transplant and Mortality in a Single-Center U.S. Cohort of Primary Sclerosing Cholangitis

**DOI:** 10.3390/jcm14062098

**Published:** 2025-03-19

**Authors:** Tamara Kahan, Ana Marenco-Flores, Natalia Rojas Amaris, Romelia Barba, Daniela Goyes, Esli Medina-Morales, Leandro Sierra, Vilas R. Patwardhan, Alan Bonder

**Affiliations:** 1 Division of Gastroenterology, Hepatology, and Nutrition, Beth Israel Deaconess Medical Center, Harvard Medical School, Boston, MA 02215, USA; tkahan@bidmc.harvard.edu (T.K.); amarenco@bidmc.harvard.edu (A.M.-F.); nrojasam@bidmc.harvard.edu (N.R.A.); vpatward@bidmc.harvard.edu (V.R.P.); 2 Department of Internal Medicine, Texas Tech University System, Lubbock, TX 79430, USA; romelia.barba@ttuhsc.edu; 3 Division of Digestive Diseases, Yale School of Medicine, New Haven, CT 06520, USA; 4 Department of Medicine, Rutgers New Jersey Medical School, Newark, NJ 07103, USA; jm2831@njms.rutgers.edu; 5 Department of Medicine, Cleveland Clinic Foundation, Cleveland, OH 44195, USA

**Keywords:** Mayo Risk Score, primary sclerosing cholangitis, liver transplant, prognosis, risk stratification

## Abstract

**Background:** The Mayo Risk Score (MRS) predicts short-term mortality in primary sclerosing cholangitis (PSC) using the age, bilirubin, albumin, aspartate aminotransferase (AST), and variceal bleeding history. While the MRS has been validated in end-stage PSC, its ability to predict liver transplantation (LT) and outcomes in newly diagnosed patients without advanced disease remains unclear. This study evaluated the effectiveness of the MRS in predicting LT and mortality in this patient population. **Methods:** We analyzed data from 109 adults with PSC enrolled in a prospective registry (2018–2024) with ≥4 years of follow-up. Logistic regression identified the predictors of LT or death, and the model performance was assessed using the area under the receiver operating characteristic curve (AUROC). Multicollinearity was evaluated using the variance inflation factor (VIF). **Results:** Among the 109 patients (mean age 45 ± 15 years, 51% female), 85% remained alive without LT, 12% underwent LT, and 3% died over a median follow-up of 4.63 years. The MRS was significantly associated with LT or death (OR 3.08, *p* < 0.001) and demonstrated excellent predictive performance (AUROC 0.99, *p* < 0.001). The model achieved 95.45% sensitivity, 98.85% specificity, and a correct classification rate of 98.17%, supporting its clinical utility. **Conclusion:** The MRS is a robust tool for risk stratification in PSC, predicting LT and mortality. These findings highlight its broader applicability beyond end-stage PSC and underscore its potential for guiding clinical management and early intervention strategies.

## 1. Introduction

Primary sclerosing cholangitis (PSC) is a chronic cholestatic liver disease characterized by inflammation and fibrosis of the intrahepatic and extrahepatic bile ducts [1]. The clinical course of PSC is highly heterogeneous, with severe cases progressing to cirrhosis, liver failure, and an elevated risk of malignancies [2]. Notably, patients with PSC, particularly those with concomitant inflammatory bowel disease (IBD), face an increased risk of cholangiocarcinoma and colorectal cancer [2,3]. Studies from tertiary referral centers estimate a median transplant-free survival of 15 to 21 years following a PSC diagnosis, although this may be longer in population-based settings [4,5]. Currently, no treatment for PSC has been demonstrated to prevent disease progression [6].

The prevalence of PSC in North America and Europe ranges from 6 to 16 cases per 100,000 individuals, with population-based studies in North America estimating an incidence of approximately 1 case per 100,000 persons [1,7,8]. Although rare, PSC poses a substantial healthcare burden due to the lack of effective therapies and its status as a major indication for liver transplantation (LT) [9]. LT is the only established treatment shown to improve survival in PSC patients with end-stage liver disease (ESLD) [10]. Among the autoimmune liver diseases (AILDs) [11], PSC is now the leading indication for LT and ranks as the fifth most common indication for LT overall in the United States [12,13].

In this context, risk prediction models are crucial to estimating prognosis and evaluating the likelihood of future events in patients with PSC. Among these models, the Mayo Risk Score (MRS) is the most widely utilized tool for estimating the short-term (4-year) mortality risk in PSC patients [14]. However, the MRS has several notable limitations, including the variability in some of its parameters, which are prone to fluctuation, and its inability to reliably predict long-term outcomes [15]. Furthermore, since the MRS was developed in a tertiary transplant center in the United States, its use has been largely restricted to patients with ESLD. In a validation and risk stratification study by Goode et al., the MRS demonstrated prognostic value for predicting the two-year outcomes in a cohort of 1001 patients, which ultimately led to the proposal of the United Kingdom-PSC (UK-PSC) score as an alternative [16].

More recently, intensive efforts have been devoted to improving prognostic modeling. While these efforts have advanced the field, distinguishing factors associated with early, rapidly progressing disease from those indicative of advanced stages remains a critical challenge [17]. A systematic review by Schmeltzer et al. compared the revised MRS with three other prognostic models: the Amsterdam–Oxford Model (AOM), the UK-PSC, and the PSC Risk Estimate Tool (PREsTo). The revised MRS outperformed the AOM in c-statistics but did not meet the 0.8 threshold indicative of strong prognostic models. In contrast, with a 10-year time horizon, the UK-PSC model achieved a c-statistic exceeding 0.8. Similarly, the PREsTo model, developed using machine learning, surpassed this threshold. Unlike the revised MRS, the PREsTo model excluded advanced PSC cases and focused on hepatic decompensation as its primary endpoint [18].

Prognostic models are essential in managing PSC, helping guide patient counseling concerning disease progression, optimizing selection for LT, and stratifying participants in clinical trials of novel therapies. Ensuring these models demonstrate transferability and can be confidently applied across diverse PSC patient populations is critical. Therefore, we aimed to further validate the MRS and assess its utility in a prospective cohort of patients in the U.S. This study is the first to validate the MRS as a predictor of LT and mortality in patients with recently diagnosed PSC who do not yet show signs of advanced disease, addressing a crucial gap in its clinical application.

## 2. Materials and Methods

### 2.1. Study Population

**Figure 1:**
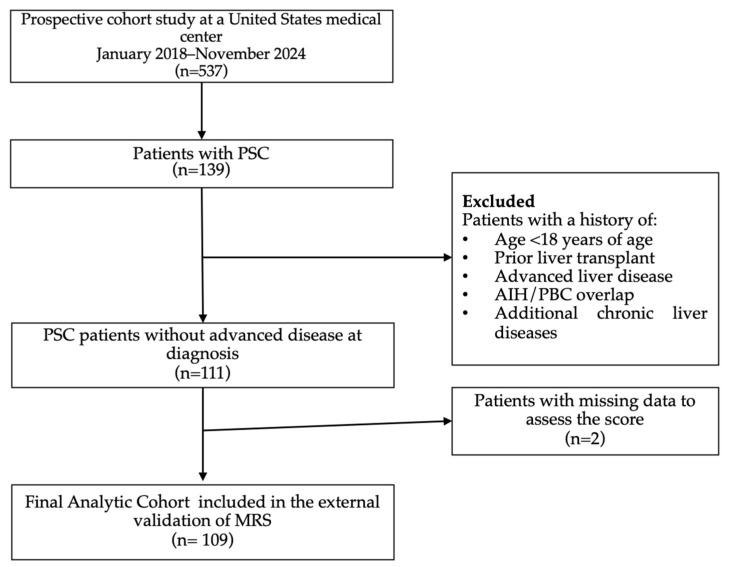
Flowchart of cohort selection. AIH: autoimmune hepatitis; MRS: Mayo Risk Score; n: number; PBC: primary biliary cholangitis; PSC: primary sclerosing cholangitis.

Participants for this study were selected from a prospective autoimmune liver disease registry at Beth Israel Deaconess Medical Center (BIDMC; Boston, MA, USA). The registry enrollment period spanned from January 2018 to November 2024. Complete follow-up was defined as LT, death, or clinical follow-up extending beyond four years. Patients who were diagnosed with PSC at the age of 18 or older, according to accepted diagnostic criteria and standards of care, were included (Figure 1).

In the early stages, PSC is often characterized by asymptomatic serum liver enzyme abnormalities, while signs of cirrhosis appear later. We established exclusion criteria to reflect cases without advanced disease, as clear clinical criteria for early-stage PSC remain challenging. To ensure this, we excluded individuals who met any of the following criteria: (a) aged under 18, (b) presence of another concurrent liver disease, (c) lack of available laboratory results, (d) cholangiocarcinoma at baseline, (e) a MELD score above 14, (f) history of liver transplantation, (g) evidence of portal hypertension at baseline, defined as the presence of varices, ascites, hepatic encephalopathy, splenomegaly, or a platelet count below 150 × 10⁹/L, (h) histologic stage 3 fibrosis, characterized by areas of fibrosis connecting to each other, or stage 4 fibrosis, marked by widespread, honeycomb-like scarring (cirrhosis), and (i) missing data required for score assessment. Ultimately, we evaluated 109 adult patients who met the cohort criteria, ensuring the selection of cases without advanced disease.

### 2.2. Study Outcomes, Variables, and Definitions

The primary outcome of this study was to assess the predictive accuracy of the MRS for LT and mortality in patients with recently diagnosed PSC who have not yet developed advanced disease. Patients were classified into three risk categories: low risk (R < 0), intermediate risk (0 ≤ R < 2), and high risk (R ≥ 2). The predicted score for each patient was calculated at baseline according to the revised MRS [14]. The MRS was determined using the following formula:

MRS = 0.03 × [age (years)] + 0.54 × In [bilirubin (mg/dL)] × In [AST (U/L)] − 0.84 × [albumin (g/dL)] + 1.24 (if a history of variceal bleeding was present)(1)

### 2.3. Statistical Analysis

Demographic, clinical, and biochemical markers were collected. Normally distributed data are reported as the mean and standard deviation (SD), while skewed data are summarized as the median and interquartile range (IQR). Continuous variable distributions were visualized using boxplots, and group comparisons were performed with *t*-tests or Mann–Whitney U tests, as appropriate. Categorical variables, expressed as percentages, were compared using Pearson’s chi-squared test (χ^2^).

Logistic regression models were used to assess factors associated with the risk of transplantation or death at diagnosis. The univariate analysis included clinically relevant variables not inherently part of the MRS estimation, including gender, presence of IBD, bile duct involvement, ursodeoxycholic acid (UDCA) treatment, and laboratory results for alkaline phosphatase (ALP), platelet count (PL), and the value of the MRS. The results are reported as odds ratios (ORs) with 95% confidence intervals (CIs). The multivariate model was constructed using stepwise logistic regression to identify the most significant independent predictors. Statistical significance was set at *p* < 0.05. Multicollinearity among the predictors in the multivariable model was assessed using the variance inflation factor (VIF).

The model’s predictive performance was evaluated by calculating and plotting the area under the receiver operating characteristic curve (AUROC) and estimating the corresponding 95% CI. Similarly, we assessed the clinical performance of the MRS by calculating the sensitivity, specificity, positive predictive value (PPV), and negative predictive value (NPV) to determine its ability to identify patients at high risk of transplant or death in PSC without advanced disease.

All the statistical analyses were conducted using Stata version 18.0 (StataCorp LP, College Station, TX, USA).

## 3. Results

### 3.1. Baseline Characteristics

The baseline characteristics of the cohort (*n* = 109) revealed a mean age at diagnosis of 45 years (SD 15), with a slight female predominance (51%). The majority of patients were White (79%), followed by African American (9%) and Hispanic (6%) patients. Concomitant IBD was present in 57% of patients, predominantly ulcerative colitis (UC) (45%), with Crohn’s disease (CD) observed in 11%. Large duct involvement was identified in 51% of patients, while small duct involvement was present in 26%. UDCA treatment was used by 57% of the cohort, with a median dose of 13 mg/kg/day. The laboratory values at diagnosis showed elevated ALP levels (median 154 IU/L, IQR 100–301) and normal TBIL levels (median 0.5 mg/dL, IQR 0.4–0.8).

The cohort was followed for a median of 4.63 years (SD 3.58), during which 85% of patients remained alive without LT, 12% underwent LT, and 3% died. The median MRS was −0.5 (IQR −1.03 to −0.02), with 80% classified as low risk (MRS < 0), 18% as intermediate risk (0 ≤ MRS < 2), and 3% as high risk (MRS ≥ 2). These findings highlight the predominance of low-risk patients at baseline, although a subset progressed to severe outcomes. The baseline characteristics of the cohort are detailed in Table 1.

### 3.2. Risk Classification

Patients were classified into the low-risk (*n* = 87), intermediate-risk (*n* = 19), and high-risk (*n* = 3) groups based on the MRS (Table 2). Concomitant IBD was observed in 45% of low-risk, 53% of intermediate-risk, and 33% of high-risk patients (*p* = 0.873). Notably, large duct involvement was more frequent in the intermediate-risk group (68%) compared to the low-risk (47%) and high-risk (33%) groups; however, this difference did not reach statistical significance (*p* = 0.177).

The laboratory markers at diagnosis varied significantly between the groups, with ALP (*p* = 0.010), AST (*p* = 0.003), ALT (*p* = 0.028), and TBIL (*p* < 0.001) all being higher in the high-risk group. At the same time, ALB was significantly lower (*p* < 0.001) in this group. The median follow-up duration was significantly shorter in the high-risk group (2.72 years) compared to the low- and intermediate-risk groups (5 years; *p* < 0.001).

The outcomes at follow-up showed that all the high-risk patients either underwent LT (33%) or died (66%), while 91% of the low-risk and 74% of intermediate-risk patients remained alive without LT (*p* < 0.001). The MRS increased progressively from the low- to high-risk groups (−0.8, 0.64, and 2.03, respectively; *p* < 0.001)

The univariate and multivariate logistic regression analyses identified key factors associated with the risk of LT or death in patients diagnosed with PSC (Table 3). In the univariate analysis, the significant predictors included the PL (OR 0.99, 95% CI 0.98–0.99, *p* = 0.031) and MRS (OR 3.00, 95% CI 1.71–5.60, *p* < 0.001) values. Additionally, the pseudo-R^2^ values indicate that the MRS (0.178) had the strongest association with transplantation or death, followed by the PL value (0.111), while the ALP value (0.013) contributed minimally to the model. Multivariate analysis confirmed that the MRS (OR 3.08, 95% CI 1.71–5.58, *p* < 0.001) was an independent predictor of adverse outcomes.

Furthermore, a multicollinearity assessment of the predictors revealed a mean VIF of 1.17, with individual values ranging from 1.05 to 1.33. This indicates no significant collinearity among the included variables. Supplementary Table S1 provides further details.

**Figure 2:**
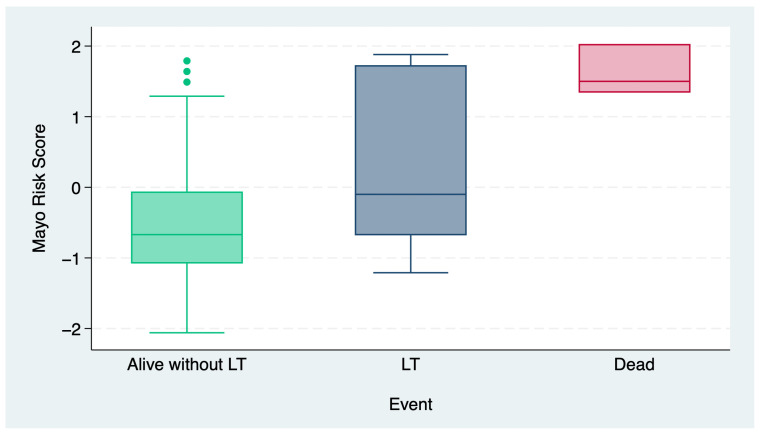
Distribution of Mayo Risk Scores across event outcomes. LT: liver transplantation (*p* < 0.001).

Figure 2 illustrates the distribution of the MRSs across the three outcome categories: alive without LT, undergoing LT, and death. Patients who remained alive without undergoing LT had the lowest MRSs, with a median score below zero and a few outliers with higher values. In contrast, patients who underwent LT exhibited elevated MRS values. The highest scores were observed in patients who died, with minimal variability within this group. These findings suggest that elevated MRS values are strongly associated with adverse outcomes, including the need for LT and mortality.

### 3.3. Predictive Performance

**Figure 3:**
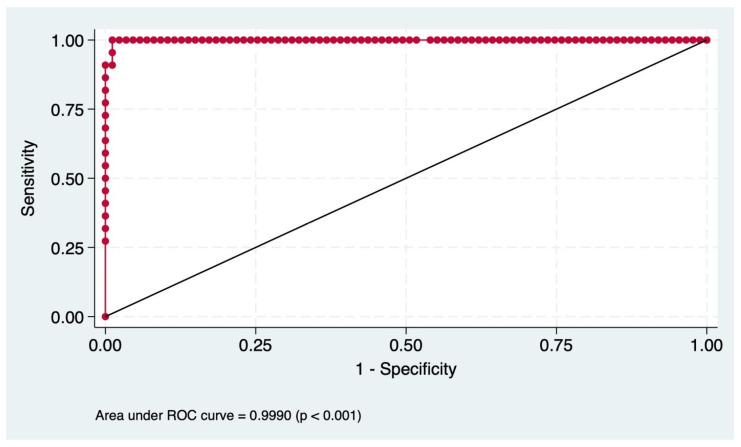
Predictive performance of the Mayo Risk Score (AUROC 0.99, *p* < 0.001). ROC: receiver operating characteristic curve. Red dots represent threshold points on the ROC curve.

We confirmed the high discriminatory power of the MRS in predicting the risk of LT and liver-related death in patients with PSC, achieving an AUROC of 0.99 (*p* < 0.001) (Figure 3).

The MRS demonstrated high predictive performance in identifying PSC patients at risk of transplant or death. With a sensitivity of 95.45% and a specificity of 98.85%, the score effectively distinguishes high-risk patients while minimizing false positives. The PPV (95.45%) and NPV (98.85%) underscore its clinical utility in accurately stratifying patients based on prognosis. The overall correct classification rate of 98.17% further supports the significant predictive accuracy of the MRS in this PSC cohort (Table 4).

## 4. Discussion

Our study demonstrates that the revised MRS is a reliable tool for predicting LT and mortality in patients with PSC. Wiesner et al. (1989) introduced the Natural History Model for PSC based on a cohort of 174 Mayo Clinic patients, identifying age, serum bilirubin, hemoglobin levels, IBD status, and histologic stage on liver biopsy as independent predictors of survival [19]. This model effectively stratified patients into the low-, moderate-, and high-risk groups. Kim et al. (2000) [14] subsequently proposed and validated the revised MRS, emphasizing its utility for risk stratification in advanced PSC [20]. Our findings build upon this work by confirming the MRS’s strong predictive performance in PSC patients without signs of advanced disease, highlighting its potential to guide clinical management before significant disease progression.

The validity of a prognostic model is best assessed in an independent yet comparable cohort. Using a prospective cohort of PSC patients enrolled at diagnosis, our study predominantly included low-risk patients based on the MRS classification. Over a five-year follow-up period, most patients survived without LT, while a subset required LT or died. The significant predictors of LT or death included a higher MRS, a severe MRS classification, and a lower ALB level, all confirmed by multivariate analysis. The MRS distribution aligned with the outcomes, with the lowest MRS among patients who survived without LT, intermediate values in those undergoing LT, and the highest in those who died. The model demonstrated excellent predictive performance (AUROC 0.99), surpassing prior studies reporting AUROCs of 0.63–0.85 [21].

The clinical impact of a recent PSC diagnosis as a predictor of LT and mortality in our study differed notably from the original MRS study, which used the date of referral rather than that of diagnosis as the starting point [14]. The MRS estimates the time to all-cause mortality but does not account for the time to LT, and for LT recipients, survival projections are based on the estimated life expectancy without LT [14]. A study by Tischendorf et al. analyzed 273 German PSC patients over a median follow-up of 76 months and found that the median survival rates based on death or LT as endpoints might be underestimated due to a high proportion (40%) undergoing LT, reflecting the hospital’s role as a leading LT center and advancements in LT techniques during 1990–2004 [21]. Similarly, Kim et al. compared the Child–Pugh classification and MRS in assessing PSC survival, noting that among 173 patients in the original MRS model, 147 had sufficient data for Child–Pugh scoring. Their findings highlighted the MRS’s superior utility in patients with less advanced disease, making it particularly valuable in clinical trials, where participants often have well-compensated liver function [22].

The MRS does not account for the variability in ALP levels, a key biochemical marker in PSC. Our study showed significantly higher baseline ALP levels in the high-risk group (*p* = 0.010). Elevated ALP, often accompanied by AST and ALT levels elevated to 2–3 times the upper limit of normal (ULN), is a hallmark of PSC and is frequently used as an endpoint in drug trials despite its predictive limitations [23]. A study by de Vries involving 366 PSC patients demonstrated the prognostic value of ALP levels, both at diagnosis and one year post-diagnosis, identifying an optimal one-year threshold of 1.3 times ULN. Notably, the one-year ALP levels were more predictive of outcomes such as LT and PSC-related deaths than the baseline levels [24]. These findings highlight the importance of developing prognostic models that integrate additional biochemical variables, particularly during follow-up, to improve the assessment of disease progression.

In addition, the ALB levels are part of the assessment of the MRS, which indicates their importance in evaluating disease severity and prognosis. However, the limited sensitivity of ALB in detecting mild or non-advanced disease reduces its effectiveness in such instances [25]. Moreover, lower pre-transplant albumin levels in patients with PSC have consistently been associated with poorer post-transplant outcomes [21,25]. Conversely, TBIL, like albumin, is included in the MRS, and elevated bilirubin remains a well-established predictor of a worse prognosis and a key component of several validated clinical predictive scores [14,19,25,26,27].

Our study found no significant differences in outcomes based on gender or concomitant IBD. While some evidence suggests that female sex and Crohn’s disease may be associated with a more favorable prognosis in PSC, this has been supported by multiple studies. A large observational study involving 44 hospitals across a geographically defined region in the Netherlands provided some of the strongest evidence for this association [4]. However, the impact of IBD on survival remains unclear, with inconsistent findings reported in the literature [28,29]. Our sample size of 109 patients was likely underpowered to detect such associations. Future studies with larger, well-adjusted cohorts are necessary to validate these findings, with a focus on the accurate classification of PSC-associated IBD and its correlation with risk stratification.

The clinical implications of our findings are significant. Early identification of high-risk patients through the MRS can inform closer monitoring, timely LT referral, and prioritization for inclusion in clinical trials. This aligns with observations by Trivedi et al., who emphasized the importance of early risk stratification in optimizing outcomes and resource allocation for PSC patients [4]. Moreover, identifying low-risk patients who are unlikely to experience rapid disease progression could reduce unnecessary interventions, alleviating healthcare costs and patient burdens.

This study’s prospective design has notable limitations, primarily due to its small cohort size. Due to the rarity of the disease, it is challenging to conduct large-scale, single-center studies on PSC. The exclusion of patients with incomplete data may have introduced selection bias, and other clinical events could impact the reliability of the risk score. Unmeasured confounders also limit the model’s applicability. Also, the single-center approach raises concerns about overfitting. Multi-center studies are needed to confirm the MRS’s broader applicability.

Additionally, while our findings affirm the predictive validity of the MRS, they do not assess its calibration. Despite these limitations, our study highlights the critical need for prolonged observation to deepen the understanding of PSC. Future research should focus on validating the prognostic factors using both retrospective and prospective methodologies and exploring their integration into novel predictive models.

## 5. Conclusions

Our findings indicate that the MRS is a reliable predictor of LT and mortality in patients with recently diagnosed PSC who have not yet progressed to the later stages of the disease. These results address a significant limitation of previous studies that primarily focused on patients with advanced-stage PSC at the baseline assessment. With excellent predictive performance and significant associations with adverse outcomes, the MRS provides a valuable tool for risk stratification in this population. These results support the broader applicability of the MRS beyond end-stage PSC and highlight its potential to guide clinical decision-making and early intervention strategies.

## Figures and Tables

**Table 1:** Baseline demographic and clinical characteristics of the study population (*n* = 109).

Variable	Baseline (*n* = 109)
Age at diagnosis, mean (SD)	45 (15)
Gender, female *n* (%)	56 (51)
Race/ethnicity, *n* (%)	
White Caucasian	86 (79)
Hispanic	7 (6)
Asian	2 (2)
African American	10 (9)
Indian American	1 (1)
Unknown	3 (3)
BMI, mean (SD)	27 (6)
Concomitant IBD, *n* (%)	
Ulcerative colitis (UC)	50 (45)
Crohn’s disease (CD)	12 (11)
Bile duct involvement, *n* (%)	
Small duct	28 (26)
Large duct	55 (51)
Dominant stricture	26 (24)
UDCA treatment, *n* (%)	61 (57)
UDCA dose, mg/kg/day	13 (4)
Liver biopsy at diagnosis, *n* (%)	45 (41)
Laboratory values at diagnosis, median (IQR)	
ALP	154 (100–301)
AST	40 (23–67)
ALT	49 (25–88)
TBIL	0.5 (0.4–0.8)
ALB	4.3 (4–4.6)
PL	265 (208–338)
INR	1 (1–1.1)
CR	0.8 (0.7–0.9)
WBC	6.6 (5.3–8.05)
Median follow-up, years mean (SD)	4.63 (3.58)
Outcome at follow-up, *n* (%)	
Alive without transplant	93 (85)
Liver transplant	13 (12)
Death	3 (3)
MRS, median (IQR)	−0.5 (−1.03–−0.02)
Risk classification Mayo Score, *n* (%)	
Low (R < 0)	87 (80)
Intermediate (0 ≤ R < 2)	19 (18)
High (R ≥ 2)	3 (3)

ALB: albumin, ALP: alkaline phosphatase, ALT: alanine aminotransferase, AST: aspartate aminotransferase, BMI: body mass index, CD: Crohn’s disease, CR: creatinine, IBD, intestinal bowel disease, IQR: interquartile range, MRS: Mayo Risk Score, PL: platelet, R: risk, SD: standard deviation, TBIL: total bilirubin, UC: ulcerative colitis, UDCA: ursodeoxycholic acid, WBC: white blood cell.

**Table 2:** Patient characteristics and outcomes based on the Mayo Risk Score classification (*n* = 109).

Variable	Low Risk (*n* = 87)	Intermediate Risk (*n* = 19)	High Risk (*n* = 3)	*p* Value
Age at diagnosis, mean (SD)	46 (15)	45 (15)	59 (23)	0.281
BMI, median (IQR)	25.1 (23–30)	26.8 (25–30)	27 (20–29)	0.395
Concomitant IBD, *n* (%)				0.873
Ulcerative colitis (UC)	39 (45)	10 (53)	1 (33)	
Crohn’s disease (CD)	10 (11)	2 (11)	0	
Bile duct involvement, *n* (%)				0.177
Small duct	24 (28)	2 (11)	1 (33)	
Large duct	41 (47)	13 (68)	1 (33)	
Dominant stricture	22 (25)	3 (16)	1 (33)	
UDCA treatment, *n* (%)	50 (82)	9 (15)	2 (3)	0.649
UDCA dose, mg/kg/day median (IQR)	13 (11–15)	12 (11–14)	12 (11–13)	0.583
Liver biopsy at diagnosis, *n* (%)	33 (38)	10 (53)	2 (67)	0.331
Laboratory values at diagnosis, median (IQR)				
ALP	139 (90–250)	293 (116–555)	268 (218–608)	0.010
AST	34 (22–57)	75 (40–116)	93 (63–132)	0.003
ALT	41 (23–74)	83 (45–127)	98 (27–192)	0.028
TBIL	0.5 (0.4–0.7)	0.6 (0.9–1.7)	1.5 (0.8–16.7)	<0.001
ALB	4.4 (4–4.7)	4 (3.8–4.4)	3.5 (3.5–3.6)	<0.001
PL	269 (214–357)	236 (157–349)	177 (163–306)	0.379
INR	1.1 (1–1.1)	1 (1–1.1)	1.2 (1–1.4)	0.636
CR	0.8 (0.7–0.9)	0.8 (0.7–0.9)	0.7 (0.6–1)	0.561
WBC	7.1 (5.3–8.4)	6 (5.4–6.6)	4.7 (3.4–7.6)	0.223
Median follow-up, years mean (SD)	5 (4.74–5)	5 (4.49–5)	2.72 (0.83–2.98)	<0.001
Outcome at follow-up, *n* (%)				<0.001
Alive without LT	79 (91)	14 (74)	0	
LT	8 (9)	4 (18)	1 (33)	
Death	0	1 (6)	2 (66)	
MRS, median (IQR)	−0.8 (−1.18-−0.26)	0.64 (0.2–1.64)	2.03 (1.85 −2.03)	<0.001

ALB: albumin, ALP: alkaline phosphatase, ALT: alanine aminotransferase, AST: aspartate aminotransferase, BMI: body mass index, CD: Crohn’s disease, CR: creatinine, IBD, intestinal bowel disease, IQR: interquartile range, LT: liver transplantation, MRS: Mayo Risk Score, PL: platelet, SD: standard deviation, TBIL: total bilirubin, UC: ulcerative colitis, UDCA: ursodeoxycholic acid, WBC: white blood cell.

**Table 3:** Univariate and multivariable logistic regression analysis for factors at diagnosis associated with the risk of transplantation or death.

Variable	Univariate OR (95% CI)	*p* Value	Pseudo R2	Variable	Multivariate OR (95% CI)	*p* Value	Pseudo R2
Gender	0.37 (0.12–1.16)	0.089	0.034				
Concomitant IBD	1.41 (0.65–3.10)	0.385	0.008				
Bile duct involvement, small duct	0.84 (044–1.61)	0.610	0.003	MRS	3.08 (1.71–5.58)	<0.001	
UDCA treatment	0.98 (0.34–2.88)	0.984	<0.001				0.1783
ALP	1.00 (0.99–1.00)	0.283	0.013				
PL	0.99 (0.98–0.99)	0.031	0.111				
MRS	3.00 (1.71–5.6)	<0.001	0.178				

ALP: alkaline phosphatase; IBD: inflammatory bowel disease; MRS: Mayo Risk Score; PL: platelet; UDCA: ursodeoxycholic acid.

**Table 4:** Performance of the Mayo Risk Score in predicting transplant or death in primary sclerosing cholangitis patients without advanced disease.

Metric	Value (%)
Sensitivity	95.45
Specificity	98.85
PPV	95.45
NPV	98.85
Correct Classification Rate	98.17

NPV, negative predictive value; PPV: positive predictive value.

## Data Availability

The data can be obtained from the corresponding author upon reasonable request.

## Supplementary Materials

The following supporting information can be downloaded at: https://www.mdpi.com/article/10.3390/jcm14062098/s1, Table S1: Variance inflation factor (VIF) for multivariate model.

## Abbreviations

The following abbreviations are used in this manuscript:

AIH	Autoimmune Hepatitis
AILDs	Autoimmune Liver Diseases
ALB	Albumin
ALP	Alkaline Phosphatase
ALT	Alanine Aminotransferase
AOM	Amsterdam–Oxford Model
AST	Aspartate Aminotransferase
AUROC	Area Under the Receiver Operating Characteristic Curve
BIDMC	Beth Israel Deaconess Medical Center
BMI	Body Mass Index
CD	Crohn’s Disease
CI	Confidence Interval
CR	Creatinine
ESLD	End-Stage Liver Disease
IBD	Inflammatory Bowel Disease
IQR	Interquartile Range
IRB	Institutional Review Board
LT	Liver Transplant
MELD	Model for End-Stage Liver Disease
MRS	Mayo Risk Score
NPV	Negative Predictive Value
PL	Platelet
PPV	Positive Predictive Value
OR	Odds Ratio
PREsTo	Primary Sclerosing Cholangitis Risk Estimate Tool
PSC	Primary Sclerosing Cholangitis
R	Risk
SD	Standard Deviation
TBIL	Total Bilirubin
UC	Ulcerative Colitis
UDCA	Ursodeoxycholic Acid
ULN	Upper Limit of Normal
UK-PSC	United Kingdom-PSC
VIF	Variance Inflation Factor
WBC	White Blood Cell
