# Supplementary material for: Performance of the Mayo Risk Score in Predicting Transplant and Mortality in a Single-Center U.S. Cohort of Primary Sclerosing Cholangitis
Source: J Clin Med. 2025 Mar 19;14(6):2098. doi: 10.3390/jcm14062098 (PMC11942813; doi:10.3390/jcm14062098)
Supplement: Supplementary file 1 [file jcm-14-02098-s001.zip › jcm-3457077-supplementary.pdf]

Table S1. Variance inflation factor (VIF) for multivariate model.

Variable	VIF	1/VIF
ALP	1.33	0.75
MRS	1.31	0.76
PL	1.14	0.88
Bile duct involvement	1.09	0.91
UDCA	1.09	0.92
Concomitant IBD	1.05	0.95
Mean VIF	1.17	

ALP: alkaline phosphatase; IBD: inflammatory bowel disease; MRS: Mayo Risk Score; PL: platelets; UDCA: ursodeoxycholic acid.
